# Supplementary material for: In silico analysis of local RNA secondary structure in influenza virus A, B and C finds evidence of widespread ordered stability but little evidence of significant covariation
Source: Sci Rep. 2022 Jan 10;12:310. doi: 10.1038/s41598-021-03767-x (PMC8748542; doi:10.1038/s41598-021-03767-x)
Supplement: Supplementary file 1 — Supplementary Information. [file 41598_2021_3767_MOESM1_ESM.zip › Supplemental Files/Supplemental File 3.zip!/Supplemental File 3/CM-AA-rev/pdf/IAV_seg6_4_1.fold.R2R.sto.pdf]

IAV_seg6_4_1

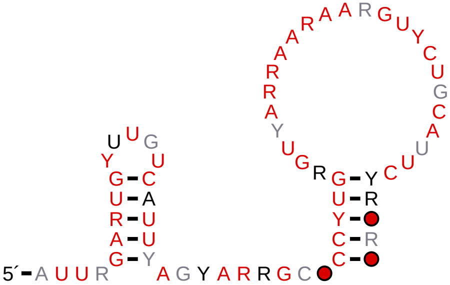

A A R
R G U
A Y
A C
R U
R G
A C
Y A
U U
U U G U
Y U G R C U
G C G Y
U A U R
R U Y
A U C R
G Y C
5´ A U U R A G Y A R R G C
